# The efficacy of single pharmacist medication review among type II diabetic patients who take six chronic medications or more: a case–control study

**DOI:** 10.1186/s13584-022-00513-0

**Published:** 2022-03-03

**Authors:** Danielle Harmatz, Shlomo Vinker, Talia Wagner, Tal Raveh, Eugene Merzon, Avivit Golan Cohen

**Affiliations:** 1 Department of Family Medicine, Sackler Faculty of Medicine, Tel Aviv University, Tel Aviv, Israel; 2 Leumit Health Services, Tel Aviv, Israel

**Keywords:** Pharmacist consultant, Polypharmacy, Chronic diseases, Diabetes mellitus

## Abstract

**Background:**

Pharmacist medication review has been implemented in many health organizations throughout the world in an attempt to alleviate the underlying risk of polypharmacy in elderly patients. These consultations are often frequent and prolonged, and are thus associated with increased costs. To date, data regarding the most effective way to utilize pharmacist consultations for the improvement of health status is scant.

**Aim:**

To evaluate the effectiveness of a single pharmacist consultation on changes in chronic medication regimes and on selected outcomes of diabetes 1-year after the consultation.

**Methods:**

A case–control study included an intervention group of 740 patients who had pharmacist consultations and a reference group of 1476 matched patients who did not have a pharmacist consultation. 1-year outcome measures were compared including changes in medications, improved safety, and objective variables such as Hba1c, blood pressure, and lipid profile.

**Results:**

In the pharmacist consultation group, there were significantly more treatment changes ([mean 1.5 vs. 0.7, *p* < 0.001 medications were stopped], and [mean 1.3 vs. 0.4, *p* < 0.05 medications were started]). Patient safety improved with a general reduction in opiates and benzodiazepines ([50.0% vs. 31.6%, *p* < 0.05 opioids were stopped] and [58.8% vs 43.8%, *p* < 0.001 benzodiazepines were stopped]). Sulfonylurea treatment reduced (10.7% vs. 3.6%, *p* < 0.05 patients who stopped Sulfonylurea) and Glucagon-like peptide-1 receptor agonists (GLP-1) increased (16.4% vs. 11.2%, *p* < 0.001 patients who started GLP-1). Additionally, HbA1c levels showed a small decrease in the pharmacist consultation group ([− 0.18 ± 1.11] vs. [− 0.051 ± 0.80], *p* = 0.0058) but no significant differences were found regarding blood pressure or lipids profile.

**Conclusion:**

A single pharmacist consultation beneficially impacted specific clinical and patient safety outcomes. Pharmacist consultations may thus help resolve polypharmacy complexities in primary care.

## Introduction

As the general population is getting older, the prevalence of co-morbid chronic diseases is increasing, leading to polypharmacy. These phenomena are of concern, as the dangers of polypharmacy include treatment errors, high prevalence of drug interactions, poorly indicated drug prescriptions, and undesired side effects [1, 2].

To address this issue, health authorities in the USA adopted the recommendations of the Agency for Healthcare Research and Quality (AHRQ) and began providing pharmaceutical consulting services to high-risk elderly patients with polypharmacy [3]. This service is widely accepted in many healthcare organizations worldwide. The Israel Ministry of Health has issued guidelines for annual review of medications in elderly patients, and encourages pharmaceutical consultation by pharmacists [4, 5].

According to the American Pharmacology Department, pharmaceutical consultation services are provided by pharmacists and include revision of prescriptions, medical-drug history, treatment indications, treatment adherence, side effects, and other factors that may influence treatment. Based on this paradigm, a program is offered to improve adherence with accepted guidelines, and for better match with patient’s preferences, to ensure optimal health, safety, and adherence [3]. The program is usually presented to the patient in a designated visit, however further visits are sometimes necessary for follow up on program adherence and further adjustments to the treatment plan [6]. A critical componentof such programs is the involvement of the patient’s family physician, though this step is not included in all programs.

The consultation process is time consuming, and its integration to ongoing care depends on available resources. It appears that despite many published studies evaluating this process, the benefits of pharmaceutical consultation have yet to be definitively proven [7–16]. The lack of clarity is related to the great variability in the implementation of the pharmaceutical consultation service. It is also linked to methodological limitations in most of the studies. Follow-up was usually short (only several months), and most studies were conducted in unique settings, focused on disease management of a specified disease and with limited outcome evaluation [8–11]. The outcome measures mostly included changes in the number of medications and adherence, usually without evaluating adherence to guidelines, impact on morbidity, and healthcare resource utilization [12–14].

A review citing over 80 studies and previous reviews, evaluated the effect of a short pharmacist consultation intervention on a wide variety of outcomes [17]. This comprehensive study represents the ongoing debate regarding the benefits of pharmacists' consultations. Beneficial effects on a variety of outcomes related to medication consumption (such as number of medications or side effects) were demonstrated, but only minimal effects on clinical outcomes (disease management or morbidity reduction), with inconsistent effects on outcomes related to health resource utilization (hospitalizations, medical encounters, emergency room (ER) visits, healthcare costs, etc.), and no effect on quality of life were found.

Leumit Health Services is one of the four health maintenance organizations in Israel and provides services to around 730,000 members nationwide. Leumit introduced short planned pharmacist consultations for patients who use a minimum of six chronic medications.

The aim of the current study was to evaluate the effectiveness of a one-time pharmacist consultation on changes in medication regimes and on outcomes of patients with diabetes, 1-year after the consultation. Findings will hopefully fill the literature gap regarding the long-term consequences of pharmacist consultation interventions.

## Methods

A case–control study was conducted, to follow outcomes of patients, who had a pharmacist consultation as compared to the outcomes of 1:2 matched controls who had no pharmacist consultations. The study took place in Leumit Healthcare Service clinics. Nationwide Leumit Healthcare clinics offer the same services provided by physicians and nurses as well as pharmacies.

*Intervention*: We introduced a nationwide project of pharmacist medication review to patients with polypharmacy and recruited pharmacists from our pharmacies. The regional pharmacist and regional medical officer selected the teams for the project. In these pre-selected healthcare centers, the pharmacists received a comprehensive training program for effective and professional patient medication review (“Appendix 1”), and the rest of the medical staff received a short introduction session to the program, at the end of which they agreed to join the project. Eligible patients were selected from the database of Leumit Health Services. At that time, projects involving the management of diabetes were the focus of the organization, thus priority had been given to patients with diabetes. According to the project design (“Appendix 2”), the patients (and their caregivers—if needed) were invited to a meeting with the pharmacist, which took place outside the regular working hours. The pharmacist evaluated the patient’s medical record before the consultation. The patients were instructed to bring all their medications to the consultation. During the consultation, after a thorough inquiry, the patient was given preliminary general recommendations, and a report with specific recommendations for the family physician. A discussion was later held with the pharmacist and the physician, after which the physician decided which of the recommendations to adopt. In the next step, the physician met the patient and implemented the changes. As the last step, a final meeting with the consulting pharmacist and the patient was held, during which the patient received an explanation and written summary containing guidelines for updated treatment.

To achieve standardization, a uniform work protocol was implemented, which included the use of five forms: a form for collecting information from the patient, a form for reviewing medicines by a pharmacist (medicines, herbs, supplements), a form for treatment recommendations for the doctor, a list of medicines and how to use them for the patient and a follow-up form.

The average length of consultation, including all stages was about four hours of pharmacist's work in the begging of 2016. As time passed, the consultant's skill improved, and the duration of the consultation was shortened to about two hours.

*Intervention group*: Patients who were not members of Leumit Healthcare Services during the 3 months prior to the consultation or during the year after the consultation were excluded from the study. The pharmacist medication review group included 740 patients who received pharmacist medication review during 2016–2017 and fulfilled the inclusion criteria.

*Control group*: We choose a control group, at a ratio of 2:1 to the intervention group, that included 1476 patients who fulfilled the criteria for pharmacist consultation, adjusted for age, sex, socioeconomic status and diabetes, from all other health care centers that were not included in the project.

### Data collected

Demographics: For each patient, the following information was retrieved: age, sex, and socioeconomic status (a continuous measure reflecting socioeconomic status based on geo-statistical affiliation of the home address as determined by the Central Bureau of Statistics in Israel, whereby 1 represents the lowest level and 20 represents the highest level) [18].

Clinical data: diagnoses of diabetes, hypertension, and hyperlipidemia, number of monthly chronic medications prescribed, adherence rate, last (before the intervention) Hba1c, last blood pressure, lipid profile, and an estimate of degree of functional handicap (handicapped was defined as a patient who is housebound according to the list of patients who receive home care).

Treatment safety: Reduction of chronic use of opiates (Anatomical Therapeutic Chemical (ATC-NO2), benzodiapines (ATC-NO5B) and in patients with diabetes, sulfonylureas (ATC-A10BB) and increase in GLP-1 (ATC-A10BJ), represented better adherence with guidelines and patient safety [2, 19–21].

Adherence with a specific medication was calculated based on the number of months of treatment during which the chronic medication was purchased in proportion to the total number of months during which the medicine was prescribed. Overall treatment adherence was calculated as the average of adherence with all chronic medications of each single patient.

We evaluated the medications that were stopped or started after the pharmacist consultation with a special focus on unsafe medications.

Outcome measures:

- Changes made in medications: number of new medications and number of medications stopped 1 year after the intervention.
- Change in adherence 1 year after the intervention in comparison to the adherence in the year before the intervention.
- HbA1c, blood pressure, and lipid profile, 1 year after the intervention in comparison to baseline measures before the intervention.
- Improvement in treatment safety; Number of Anxiolytics (ATC-NO5B) stopped, number of Anxiolytics (ATC-NO5B) started. Number of Opioids (ATC-NO2) stopped, Number of Opioids (ATC-NO2) started. Patients with diabetes who stopped Sulfonylureas (ATC-A10BB), Patients with diabetes who started Sulfonylureas (ATC-A10BB). Patients with diabetes who stopped Glucagon-like peptide-1 receptor agonist (GLP-1) (ATC-A10BJ), Patients with diabeteswho started GLP1 (ATC-A10BJ).

### Statistical analysis

Statistical analysis was performed using STATA 12.0. Assumptions were two sided with an α of 0.05. Initial analysis compared demographic characteristics between the study groups (intervention vs control), using paired t-test and Fischer exact χ^2^ test for continuous and categorical variables, respectively, based on normal distribution and variable characteristics. Categorical data are shown in counts and percentages. Data on continuous variables with normal distribution are presented as mean and 95% confidence interval (CI).

To account for effects of confounders we used multivariate logistic regression models to estimate the odds ratios (OR) and 95% CI for independent association pharmacist consultation group and the likelihood of the reduction of at least one medication.

The study was approved by the Internal Review Board of Leumit Health Services.

## Results

The study population included 2216 patients, 740 in the intervention group, and 1476 in the control group.

The demographic and clinical background data of the study population is presented in Table 1. Despite matching and minimum threshold of six chronic medications, the average number of medications was significantly higher in the pharmacist consultation group (11.4 vs 9.9, *p* < 0.05).

**Table 1:** Baseline characteristics in pharmacist consultation group and control group:

Variable	Pharmacist consultation group (N = 740)	Control group (N = 1476)	*p*-value
Age (years, Mean; SD)	70.0 ± 11.5	70.3 ± 11.6	0. 540
Female N (%)	351 (47.4)	698 (47.2)	0.950
Socio-economic status score (Mean; SD)	12.34 ± 17.5	12.5 ± 17.0	0.450
Chronic medications N (Mean; SD)	11.4 ± 2.4	9.9 ± 2.4	0.001
DM (N, %)	547 (73.9)	1092 (73.9)	0.973
HTN (N, %)	648 (87.5)	1329 (90.0)	0.074
Dyslipidemia (N, %)	645 (87.1)	1261 (85.4)	0.261
Homebound (N, %)	117 (15.8)	249 (16.8)	0.524
HbA1c* (Mean; CI)	7.21 ± 1.7	6.92 ± 1.3	0.001
SBP (mmHg)* (Mean; SD)	134.9 ± 19.3	134.3 ± 17.1	0.433
DBP (mmHg)* (Mean; SD)	68.2 + 25.1	75.50 ± 9.8	0.001
Total Cholesterol (mg/dl)* (Mean; SD)	175.6 ± 44.4	168.7 ± 37.8	0.001
TG (mg/dl)* (Mean; SD)	160.6 ± 87.9	156.0 ± 85.9	0.571
HDL Cholesterol (mg/dl) * (Mean; SD)	45.6 ± 11.8	45.4 + 12.4	0.753
LDL Cholesterol (mg/dl)* (Mean; SD)	98.7 ± 35.5	92.4 ± 31.1	0.001

DM, Diabetes Mellitus; HTN, Hypertension; SBP, Systolic Blood Pressure; DBP, Diastolic Blood Pressure; T. Cholesterol, Total Cholesterol; TG, Triglycerides

*For patients with the relevant chronic disease

In other parameters, the clinical data show some between group differences as the patients in the pharmacist consultation group had less controlled diabetes (mean HBA1c 7.2 vs 6.9, *p* < 0.05).

Data related to changes in treatment regimen in general and for selected chronic diseases, 1 year after the intervention are presented in Table 2. In the pharmacist consultation group, there were significantly more treatment changes (1.5 vs. 0.7, *p* < 0.05 medications were stopped, and 1.3 vs. 0.4, *p* < 0.05 medications were started).

**Table 2:** Changes in medication treatment 1 year after pharmacist consultation:

Variable	Pharmacist consultation group (N = 740)	Control group (N = 1476)	*p*-value
Total medications at baseline N (Mean; CI)	11.4 ± 2.4	9.9 ± 2.4	< 0.001
Medications stopped N (Mean; SD)	1.5 ± 1.1	0.7 ± 0.9	< 0.001
Medications started N (Mean; SD)	1.3 ± 0.8	0.4 ± 0.8	< 0.001
Proportion of patients that stopped at least one medication, N (%)	610 (82.4)	712 (48.2)	< 0.001
Proportion of patients that started at least one medication, N (%)	483 (65.2)	373 (25.2)	< 0.001
Medications adherence before % (Mean; SD)	79.3 ± 16.3	83.7 ± 14.3	0.014
Medications adherence after % (Mean; SD)	81.1 ± 15.1	88.4 ± 11.8	< 0.001
Difference in adherence % (Mean; SD)	1.6 ± 13.6	4.7 ± 14.2	0.036
Patients on anxiolytics N (%)	136 (18.4)	377 (25.5)	< 0.001
Patients who stopped anxiolytics (BNZ) N (% anxiolytics users)	80 (58.8)	165 (43.8)	0.029
Patients who started anxiolytics (BNZ) N (%)	30 (22)	142 (37.7)	< 0.001
Patients on chronic opioids 341 pts N (% opioids users)	72 (9.7)	269 (18.2)	< 0.001
Patients who stopped opioids N (%)	36 (50.0)	85 (31.6)	< 0.001
Patients who started opioids N (%)	22 (30.6)	114 (42.4)	0.07
Diabetic patients with sulfonylureas N (%)	112 (20.4)	252 (23.1)	< 0.001
Diabetic patients who stopped sulfonylureas, N (%)	12 (10.7)	12 (3.6)	< 0.001
Diabetic patients who started Sulfonylureas, N (%)	10 (8.9)	35 (13.8)	< 0.001
Diabetic patients with GLP1 N (%)	98 (17.9)	160 (14.6)	0.06
Diabetic patients who stopped GLP1, N (%)	2 (2.0)	15 (9.4)	< 0.001
Diabetic patients who started GLP1, N (%)	16 (16.4)	18 (11.2)	0.031

Better adherence with guidelines for patient safety was demonstrated in the pharmacist consultation group with a general reduction in utilization of opiates and benzodiazepines ([50.0% vs. 31.6%, *p* < 0.05 opioids were stopped] and [58.8% vs 43.8%, *p* < 0.05 benzodiazepines were stopped]). Among patients with diabetes there was a reduction in sulfonylurea treatment (10.7% vs 3.6%, *p* < 0.05 patients who stopped Sulfonylurea) and increase in GLP-1 (16.4% vs 11.2%, *p* < 0.05 patients who started GLP-1), representing better adherence with guidelines and with patient safety in the pharmacist consultation group. Compared to the control group, no improvement in adherence in the pharmacist consultation group was demonstrated.

After adjusting for age, sex, the total number of chronic medications, diagnosis of diabetes, hypertension, and hyperlipidemia (see Tables 3 and 4), pharmacist consultation was positively associated with implementation of changes in treatment regimens. Pharmacist consultation increased the likelihood of the addition of at least one medication [adjusted OR of 8.38 (95% CI 6.73–10.42 CI) and in the same manner the likelihood of the reduction of at least one medication [adjusted OR of 95% CI 4.54 (3.63–5.69 CI)]. (Full data not presented).

**Table 3:** Crude and adjusted ORs for addition of at least one medication in treatment regimen

Variable	Crude OR (95% CI)	*p*-value	Adjusted OR* (95% CI)	*p*-value
Pharmacist consult (Yes/No)	5.51 (4.48; 6.67)	< 0.001	8.34 (6.7; 10.4)	< 0.001
Diabetes mellitus (Yes/No)	0.81 (0.60; 1.01)	0.053	1.0 (0.8; 1.3)	0.450
Dyslipidemia (Yes/No)	0.80 (0.59; 1.01)	0.074	0.7 (0.5; 1.0)	0.084
Hypertension (Yes/No)	0.62 (0.43; 0.81)	< 0.001	0.84 (0.6; 1.1)	0.414
Age (per 1 additional year)	1		0.94 (0. 9; 1.0)	0.794
Gender (Female/Male)	1		1.01 (0.1; 1.0)	0.890
SES (per 1 additional point)	1		0.95 (0.9; 1.0)	0.770
Total № of medications (per 1 additional medication)	1		0.82 (0.8; 0.8)	< 0.001

SES, Socioeconomic status

*Adjusted for chronic diseases (DM, Hypertension, Dyslipidemia)

**Table 4:** Crude and adjusted ORs for reduction of at least one medication in treatment regimen

Variable	Crude OR (95% CI)	*p*-value	Adjusted OR* (95% CI)	*p*-value
Pharm consult (Yes/No)	5.03 (4.04; 6.29)	0.001	4.54 (3.63; 5.69)	0.001
Diabetes mellitus (Yes/No)	1.49 (1.22; 1.81)	0.004	0.99 (0.79; 1.25)	0.612
Dyslipidemia (Yes/No)	1.36 (1.06; 1.74)	0.03	1.10 (0.82; 1.56)	0.314
Hypertension (Yes/No)	1.39 (1.06; 1.85)	0.014	1.17 (0.83; 1.65)	0.359
Age (per 1 additional year)	1		0.99 (0.98; 1.01)	0.888
Gender (Female/Male)	1		0.97 (0.80; 1.18)	0.948
SES-AGAS (per 1 additional number)	1		0.99 (0.99; 1.00)	0.679
Total № of medications (per 1 additional medication)	1		1.23 (1.19; 1.27)	0.001

SES, Socioeconomic status

*Adjusted for chronic diseases (DM, Hypertension, Dyslipidemia)

Outcome measures in diabetes, 1 year after the intervention are presented in Tables 5 and 6. HbA1c showed a significant improvement in the pharmacist consultation group ([− 0.18 ± 1.11] vs. [− 0.051 ± 0.80], *p* = 0.0058). Outcome measures in hypertension and hypercholesterolemia 1 year after the intervention are presented in Table 6. Regarding hypertension and hypercholesterolemia, no significant differences between the Pharmacist consultation group and the control group in outcome measures was noted.

**Table 5:** Crude and adjusted ORs for decrease of HbA1c level among diabetic patients

Variable	Crude OR (95% CI)	*p*-value	Adjusted OR* (95% CI)	*p*-value
Pharmacist consultation	1.10 (0.90; 1.40)	0.091	1.22 (1.00; 1.50)	0.046
Age (per 1 additional year)	0.91 (0.90; 0.91)	0.048	1.01 (0.90; 1.00)	0.781
Gender (Female/Male)	1.34 (0.90; 1.70)	0.063	1.16 (0.92; 1.35)	0.089
SES (per 1 additional number)	0.92 (0.90; 0.94)	0.034	1.01 (0.96; 1.04)	0.403
Hypertension (Yes/No)	0.81 (0.52; 1.11)	0.222	0.84 (0.62; 1.21)	0.585
Dyslipidemia (Yes/No)	0.73 (0.41; 1.18)	0.314	0.82 (0.54; 1.03)	0.121
Total number of medications (per 1 additional medication)	0.90 (0.87; 0.93)	0.014	0.96 (0.89; 1.03)	0.164

SES, Socioeconomic status

*Adjusted for age, gender, SES, total number of medications and chronic diseases (DM, Hypertension, Dyslipidemia)

**Table 6:** Changes in outcome measures in chronic diseases 1 year after the pharmacist consultation

Variable	Intervention group (N = 740)	Control group (N = 1476)	*p*-value
HgbA1C %	− 0.18 ± 1.11	− 0.51 ± 0.80	0.006
SBP (mmHg) change (Mean; SD)	− 1.33 ± 20.79	− 0.83 ± 19.35	0.352
DBP (mmHg) change (Mean; SD)	− 3.05 ± 24.55	− 1.01 ± 10.95	0.0153
HDL cholesterol (mg/dl) change (Mean; SD)	− 0.43 ± 7.73	− 0.024 ± 6.86	0.241
LDL cholesterol (mg/dl) change (Mean; SD) 1906 pts	− 2.42 ± 34.27	− 0.60 ± 26.28	0.206
TG (mg/dl) change (Mean; SD)	− 1.11 ± 74.42	− 0.96 ± 71.37	0.967

SBP, Systolic Blood Pressure; DBP, Diastolic Blood Pressure; T. Cholesterol, Total Cholesterol; TG, Triglycerides

## Discussion

In this study we evaluated the effect of a single pharmacist consultation on the quality and safety of the treatment of elderly patients with diabetes regarding polypharmacy. The study included 2216 patients, 740 of them had a pharmacist consultation. Intervention and reference groups were matched for age, sex, and socioeconomic status and diagnosis of diabetes. The reasons for polypharmacy in the intervention groups included prevalence of chronic diseases (diabetes mellitus (DM), hypertension (HTN) and hyperlipidemia). There was no between group difference regarding the rate of concomitant chronic illnesses for which participants received various medications, that led to polypharmacy. To control for the effects of potential confounders, multivariate logistic regression models were employed.

This is the first large comparative study to evaluate the impact of a single pharmacist consultation based on outcome measures and alterations in medication regimens.

To date, most studies regarding impact of pharmacist consultation had no reference groups, had small patient groups, and were conducted in specific outpatient clinics or in otherwise select populations. In contrast, the current study was conducted in a “real world “ rather than a research setting, among a patient population of individuals with diabetes who were treated with polypharmacy [22–24]. In addition, aside from the intervention group that received pharmacist consultation, there was a reference group that did not receive pharmacist consultations. Moreover, the accepted definition of the nature of the intervention was formulated by the Pharmaceutical Care Network Europe in 2018 [25], precluding clear conclusions from studies published prior to 2018. New studies of various pharmacist consultation models are thus needed.

The current study demonstrated that a single pharmacist consultation was associated with significant changes in treatment regimens. In the intervention group, significantly more chronic medications were stopped, and new ones were added. These findings are in concordance with several previous studies [8, 10].

The pharmacist consultations in our study had no impact on adherence, possibly because the long-term effect of a single consultation is limited. Previous studies which evaluated the effects of multiple session interventions lasting six [7] or 9 months [26], demonstrated improvement of up to 10.9% in adherence in comparison to a reference group. However, it is important to note that this positive effect faded shortly after termination of the interventions.

The effect of the single consultation on outcome measures of patients with diabetes was significantly positive only in the reduction of HbA1c levels. These findings are similar to those observed in a previous study that specifically targeted patients with diabetes [27]. In addition, we found that treatment changes improved the use of treatment guidelines. This finding supports the hypothesis that pharmacist consultations lead to improved quality of care.

The impact of the intervention on patient safety was evaluated by analyzing the rate of unsafe medications used. One group of unsafe medications was sulfonylureas, which according to the American Diabetes Association’s type 2 diabetes guidelines, increase the risk of severe hypoglycemia and cardiovascular mortality. The FDA issued a warning regarding their use. In our study, the number of patients who stopped sulfonylureas was significantly higher, and the number of patients who started them was significantly lower in the intervention group. The second group included sedative or narcotic drugs that considered inappropriate for long term use in older adults. We found that in the intervention group, more benzodiazepines and opioids were stopped, and fewer patients were started those medications.

Our study demonstrates that a single pharmacist consultation has a positive impact on specific outcomes but does not affect adherence. We found improvements in patient safety in the management of diabetes, and chronic opioid and benzodiazepine utilization. These outcomes support this intervention as a good option for coping with the increasing challenges of polypharmacy among patients with diabetes in community primary care clinics.

### Strengths and weaknesses of the study

Our observational study compared a study group of patients that had pharmacist consultations as compared to a 1:2 matched control group that had no intervention. This study design limited our ability to form a cause-and-effect relation. Also, we cannot exclude the possibility that our results were due to a nonspecific effect of increased patient interaction in the intervention arm. Furthermore, because the intervention had several components, such as verbal counseling, followed by written materials, that were geared for individuals with probable low health literacy, we could not attribute intervention effects to any single component.

Although the project recruited patients with polypharmacy, a special focus was on diabetes. Thus, we matched the control population to have the same prevalence of diabetes. The chronic disease burden was similar in both groups in terms of the frequency of hypertension and hyperlipidemia. In the pharmacist consultation group patients had more chronic medications at baseline but after correction for the number of medications in a multivariable analysis the pharmacist consultation was still statistically significant. Other confounders might be differences in doctors' education and their motivation to implement clinical guidelines. Beginning in 2011 Leumit Health Services implemented a chronic care program to improve diabetes care in general practice: MESSAGE program (Motivation, Education, Skills and Supervision to Achieve better diabetes care in the General practice Environment), which included a training course for doctors and nurses and an ongoing time allocation for proactive diabetes care [28].

Finally, the design of the study required patients to be capable of discussing their medications regimen and provide accurate information regarding their adherence. Hence, they were more aware of their treatment than patients in general, which may create selection bias towards patients who demonstrate better insight regarding their pharmacotherapy than the general population of patients.

The study has internal validity because it was based on a validated database of medication purchases, chronic diagnoses and laboratory tests that was similar for the pharmacist consultation group and the control group. As for external validity, because the study was done with only one healthcare organization, it is possible that the indications for referral to pharmacological consultation, as well as the recommendations for changes in treatment regimens may be different from those in other healthcare organizations. However, our patients are distributed nationwide so this study is representative of the country’s population.

In conclusion, we demonstrated the beneficial effect of a single pharmacist consultation on medication regimens and patient safety and contributed to better management of diabetes t. We strongly recommend adopting this method of consultation that allows for relatively simple and affordable widespread implementation.

## Data Availability

The datasets generated and analyses during the current study are not publicly available due to individual privacy but are available from the corresponding author on reasonable request.

## Appendix 1

### Syllabus of training program for the consulting pharmacists (intervention group)

**Table:**

Topic	Teaching method	Duration of the study unit
Introducing the principles of the pharmaceutical consulting model	Workshop	6 Hours
Drug-drug interactions	Lecture	3 Hours
Principles of using old and new oral anticoagulants	Lecture	3 Hours
Polypharmacy in the elderly	Lecture	3 Hours
Use of databases for pharmacological consulting	Workshop	3 Hours + 8 Hours
A school for the practice of treating hypertension	Combined methods	9 Hours (3 sessions)
A school for the practice of treating Diabetes	Combined methods	15 Hours (5 sessions)
Treatment of supra ventricular arrhythmias	Lecture	3 Hours
Self-clinical experience and presentation of cases of diabetes	Workshop	3 Hours
Treatment of ventricular arrhythmias and ischemic heart disease, other heart diseases	Lecture and Case-based practice	8 Hours
Treatment of Hyperlipidemia	Lecture and Case-based practice	3 Hours

## Appendix 2

### Project design

1. *Recruitment of patients for the project*: Pharmacists' training included a variety of topics with a focus on diabetes and therefore, naturally, most of the patients recruited were diabetics.
  To identify patients in need of intervention, a report was developed that included patients consuming 6 or more medications and labeled diabetics whose HbA1C levels were unbalanced.
2. *The medication review protocol*: An organizational protocol for pro-active pharmacological consultation was developed (protocol no. 040502075146, version 1.; 1.8.2016) based on Protocol No. 113 of the Ministry of Health in Israel).
  The consultation stages, in accordance with the protocol, included:
  2.1 Reaching out of relevant patients.
  2.2 Signing the patients on consent form for counseling
  2.3 Learning of all the information that appears in the patient's medical record: the patient's diagnoses, the correlation of the drugs with the diagnoses, testing of therapeutic effectiveness in accordance with laboratory tests and other clinical indicators.
  2.4 Identify drug-related issues that require intervention: dose adjustment to renal function tests, life-threatening drug–drug interactions, dose adjustment according to the guidelines relevant for the diagnosis, side effects of the drugs and Issues related to patient adherence to treatment.
3. *The meeting plan between the pharmacist and the patient*: Prior to the session the patient is instructed to bring his medication with him. The session itself includes an examination of the degree of response to treatment and correct adherence to the treatment instructions, an explanation of the goals of the treatment, an examination of the therapeutic effectiveness and the existence of side effects, as well as an inquiry about additional medical information that could not be traced in the medical record. Explanation on a healthy lifestyle is also part of the encounter with the pharmacist.
4. *The meeting plan between the pharmacist and the primary care physician*: The pharmacist shares his recommendations about the drug treatment and documents them in the medical records. The decision regarding the adoption of the recommendations is up to the doctor alone.
5. *Summary meeting between the pharmacist and the patient*: The pharmacist gives the patient a table with the final information about his treatment regimen and how to take it, according to the decision of his primary care physician, and makes sure that the patient understands and agrees.

The frequency of appointments depended on the availability of the physician and the patient's ability to make an appointment with the pharmacist. All the meetings took place with a gap of no more than 2 weeks.
